# The Impact of Caloric and Non-Caloric Sweeteners on Food Intake and Brain Responses to Food: A Randomized Crossover Controlled Trial in Healthy Humans

**DOI:** 10.3390/nu10050615

**Published:** 2018-05-15

**Authors:** Camille Crézé, Laura Candal, Jérémy Cros, Jean-François Knebel, Kevin Seyssel, Nathalie Stefanoni, Philippe Schneiter, Micah M. Murray, Luc Tappy, Ulrike Toepel

**Affiliations:** 1 Department of Physiology, Faculty of Biology and Medicine, University of Lausanne, 1005 Lausanne, Switzerland; crezecamille@gmail.com (C.C.); candlau@gmail.com (L.C.); jeremy.cros@unil.ch (J.C.); kevin.seyssel@unil.ch (K.S.); nathalie.stefanoni@unil.ch (N.S.); philippe.schneiter@unil.ch (P.S.); luc.tappy@unil.ch (L.T.); 2 The Laboratory for Investigative Neurophysiology (The LINE), Departments of Radiology and Clinical Neurosciences, University of Lausanne and Lausanne University Hospital, 1011 Lausanne, Switzerland; jean.francois.knebel@gmail.com (J.-F.K.); micah.murray@chuv.ch (M.M.M.); 3 Electroencephalography Brain Mapping Core, Center for Biomedical Imaging (CIBM) of Lausanne and Geneva, 1015 Lausanne, Switzerland; 4 Department of Hearing and Speech Sciences, Vanderbilt University, Nashville, TN 37232, USA; 5 Department of Ophthalmology, Jules Gonin Eye Hospital, 1004 Lausanne, Switzerland; 6 Metabolic Center, Hôpital Intercantonal de la Broye, 1470 Estavayer-le-Lac, Switzerland

**Keywords:** electroencephalography, non-nutritive sweeteners, sweet taste, visual food cues, food intake, ad libitum buffet

## Abstract

Whether non-nutritive sweetener (NNS) consumption impacts food intake behavior in humans is still unclear. Discrepant sensory and metabolic signals are proposed to mislead brain regulatory centers, in turn promoting maladaptive food choices favoring weight gain. We aimed to assess whether ingestion of sucrose- and NNS-sweetened drinks would differently alter brain responses to food viewing and food intake. Eighteen normal-weight men were studied in a fasted condition and after consumption of a standardized meal accompanied by either a NNS-sweetened (NNS), or a sucrose-sweetened (SUC) drink, or water (WAT). Their brain responses to visual food cues were assessed by means of electroencephalography (EEG) before and 45 min after meal ingestion. Four hours after meal ingestion, spontaneous food intake was monitored during an ad libitum buffet. With WAT, meal intake led to increased neural activity in the dorsal prefrontal cortex and the insula, areas linked to cognitive control and interoception. With SUC, neural activity in the insula increased as well, but decreased in temporal regions linked to food categorization, and remained unchanged in dorsal prefrontal areas. The latter modulations were associated with a significantly lower total energy intake at buffet (mean kcal ± SEM; 791 ± 62) as compared to WAT (942 ± 71) and NNS (917 ± 70). In contrast to WAT and SUC, NNS consumption did not impact activity in the insula, but led to increased neural activity in ventrolateral prefrontal regions linked to the inhibition of reward. Total energy intake at the buffet was not significantly different between WAT and NNS. Our findings highlight the differential impact of caloric and non-caloric sweeteners on subsequent brain responses to visual food cues and energy intake. These variations may reflect an initial stage of adaptation to taste-calorie uncoupling, and could be indicative of longer-term consequences of repeated NNS consumption on food intake behavior.

## 1. Introduction

Excess sugar consumption, in particular in the form of sugar-sweetened beverages (SSBs), has been repeatedly identified as a major factor contributing to weight gain, overweight and obesity prevalence as well as associated metabolic disorders [1,2,3]. In order to fight increasing rates of overweight and obesity and help body weight management, many non-caloric molecules with a high sweetening power, called non-caloric or non-nutritive sweeteners (NNS), were developed and introduced into our daily diet. That is, hedonic properties of sweet taste can be enjoyed without consuming excess liquid calories. Yet, epidemiological studies have shown a link between NNS consumption and an increased prevalence of overweight and obesity risk on the long-run [4,5,6]. Although risks of reverse causality cannot be excluded when interpreting the results of observational cohort studies, one other reason for such a link could be functional properties of the central food intake regulation system discussed below.

In healthy humans, food intake is regulated by a fine-tuned balance between drives towards palatable items (‘hedonic’ processing and reward valuation), physiological needs (‘homeostatic’ processing), and inhibitory control. While inhibitory control is supported by brain areas of the executive function network, i.e., dorsal, ventrolateral prefrontal and parietal regions [7], reward valuation is assisted by cortico-limbic networks comprising basal ganglia, the anterior cingulate and orbitofrontal cortices [8]. Reward is integrated with homeostatic signals in the hypothalamus and insula. Gastro-intestinal hormones secreted before (e.g., ghrelin) and after a meal (e.g., insulin), nervous afferents as well as sweet taste receptor activation in the mouth (and possibly along the digestive track) all provide feedbacks to the brain and thereby inform these networks on physiological states and consequences of food ingestion [9,10,11]. This regulatory system likely evolved with a caloric value assigned to sweet taste, as naturally occurring sweeteners generally contain about 4 kcal/g. Due to their sweet but non-caloric properties NNS might thus provide erroneous information to the food intake regulatory system, potentially inducing maladaptive food choices as compensatory mechanism [12,13].

In animals, findings support this hypothesis by showing detrimental effects of NNS consumption on food intake behavior and increased adiposity and body weight longitudinally [14,15]. So far, the impact of NNS consumption on food choices and body weight control in humans remains controversial, showing either beneficial, detrimental, or no effects [16,17,18,19]. From a neurophysiological perspective, some studies have shown differences in the cerebral processing of taste information between NNS and sucrose stimuli of equal sweetness intensity, highlighting the capacity of the human brain to readily discriminate between caloric and non-caloric sweet taste [20,21,22]. However, these studies assessed immediate gustatory responses and neural activity, but not subsequent food cravings and intake of excess solid calories.

Neuroimaging studies in humans have further shown that food cravings and choices often result from exposure to visual food cues [23,24]. However, only one study, to our knowledge, has investigated brain response modulations to food cue exposure following NNS consumption in humans, i.e., in the context of a 3-month replacement of sucrose-sweetened beverages by artificially sweetened equivalents [25]. This former study of our group highlighted the potential implication of central cognitive control mechanisms in weight loss failure. However, that study did not directly compare the impact of consuming caloric vs. non-caloric sweeteners on responses. Further, participants had not been blinded to the type of beverage they consumed. Thus, how NNS influence subsequent drives towards certain types of foods (in particular sweet foods) and how this relates to modulations in food intake behavior remains largely unknown.

The goal of our current study was thus to investigate whether, as compared to water consumption, activation of sweet taste receptors with NNS or with sucrose, as part of a standardized meal, exert different acute effects on (a) postprandial brain responses to food viewing, (b) postprandial gastro-intestinal hormone secretion known to impact hunger and satiety feelings and (c) subsequent food intake behavior, both in terms of quantity and quality of choices (ad libitum buffet).

## 2. Materials and Methods 

### 2.1. Participants

Eighteen healthy, normal-weight men were recruited. All volunteers used to drink on average ≥3 cans of 33 cL of SSBs per week. None of the participants had current, prior, or family history of diabetes, cardiovascular, kidney, hepatic, neurological or psychiatric disease. Further exclusion criteria were color blindness, particular diets (e.g., vegetarianism), any food intolerance or allergy, arterial blood pressure > 140/90 mmHg at rest, exercising for more than 3 h per week, current medication, drug-taking or smoking habits, and consuming more than 10 g of alcohol per day. Only infrequent consumers of NNS-sweetened beverages (≤1 can of 33 cL per week) were included. All volunteers were weight-stable, right-handed according to the Edinburgh Handedness Inventory [26], and had normal or corrected-to-normal vision. To ensure medical safety, volunteers were not included when hemoglobin and ferritin levels were below 13.5 g/L and 50 µg/L respectively, when weighing less than 50 kg, and when having donated blood or participated to another clinical trial in the prior three months.

**Figure 1:**
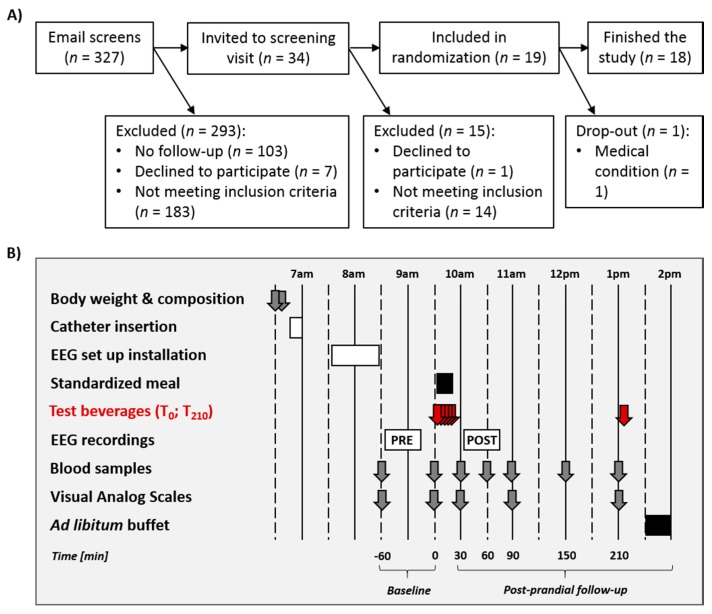
(**A**) Flow diagram for study participants’ eligibility, enrollment, and follow-up. (**B**) Detailed protocol of the in-center test days. Identical procedures were used on the three test days. Test beverages (WAT, SUC, NNS) were ingested at *T* = 0 min (350 mL; five 70 mL-glasses every 5 min) and at *T* = 210 min (one 200 mL glass). EEG: electroencephalographic recording session.

Recruitment was done by means of advertising at local university campuses. Recruitment, screening, and follow-up of participants is shown in Figure 1A. Potential participants were first screened by email and then invited to a screening visit. Nineteen volunteers met all eligibility criteria and were enrolled in the study. One participant had to be excluded due to medical discomfort during the first test day; so that 18 volunteers completed the entire protocol.

### 2.2. General Procedure

The study consisted of three in-center test days for each volunteer, on which one of the three beverage conditions (i.e., Water, Sucrose, and NNS consumption, further referred to as WAT, SUC, and NNS) was tested in a randomized crossover controlled design. The beverage conditions were separated by a wash-out period of three weeks. Each test day was preceded by a 5-day nutritional and lifestyle recommendation period followed by a 2-day run-in period. During this run-in period, volunteers received a controlled weight-maintenance diet (55% carbohydrates (including 10% as sugars), 30% lipids and 15% proteins) calculated from the Harris–Benedict equation with a physical activity factor set at 1.5. Participants were instructed to consume all meals and snacks at specified times of the day (7 a.m., 10 a.m., 12 a.m., 3 p.m., 7 p.m.), and to drink only water.

Detailed procedures for the in-center test days are provided in Figure 1B. On each test day, volunteers reported to the Metabolism, Nutrition, and Physical Activity unit from the Clinical Research Center of the Lausanne University Hospital at 6.30 a.m. They were fasting since 10 p.m. the evening before the test. They were asked to void, and body weight was measured thereafter (Seca 708, Seca GmbH, Hamburg, Germany). Body composition was assessed using bio-electrical impedancemetry (Biacorpus, Medical Healthcare GmbH, Germany). Each volunteer was then placed in a bed, and a catheter was inserted into an antecubital vein of the left forearm to allow for repeated blood collection throughout the test day. The venous path was kept open with a slow perfusion of saline solution (NaCl 0.9%). Two blood samples were collected in fasted state at *T* = −60 and 0 min before standardized meal and beverage ingestion. At those time points, participants were also asked to fill visual analog scales (VAS; 0–100) for hunger, thirst, and satiety levels as well as a Likert scale (LS; 1–9) for taste cravings. Each volunteer was then accompanied to a light-proof room where the electroencephalographic (EEG) recording system was installed. A cap with 64 active electrodes was placed on the participants’ head and prepared so that electrical impedance between electrical sensor and scalp were kept below 40 kΩ. A first EEG recording took place between *T* = −60 and 0 min (i.e., further referred to as the pre-prandial recording session), while participants completed an online continuous recognition task. The EEG recording procedure, as well as visual stimuli and task used are detailed below (Section 2.8). At *T* = 0 min, a standardized meal and the 350 mL test drink (T_0_-beverage; WAT, SUC, or NNS) were given to each volunteer. Five blood samples were collected over the post-prandial period, at *T* = 30, 60, 90, 150 and 210 min after meal and beverage consumption had started. At *T* = 30, 60 and 210 min, participants also filled VAS and LS for hunger, thirst, satiety, and taste cravings. A second EEG recording took place 45 min after meal and beverage ingestion (i.e., further referred to as the post-prandial recording session), that followed the same procedure as the pre-prandial session. In order to avoid ceiling effects and further strengthen the impact of beverage type on spontaneous food intake, participants received a 200 mL pre-buffet drink 210 min after the meal ingestion (T_210_-beverage; SUC, WAT or NNS), its composition repeating the beverage condition. This drink served as a preload for further quantitative and qualitative assessments of spontaneous food intake at an ad libitum buffet taking place 20 min after the preload ingestion.

The primary study outcomes were pre- to post-prandial changes in brain responses to food viewing across beverage conditions. All behavioral and physiological parameters were considered as secondary outcomes. Study sample size was determined by assuming the same effect size on the spatio-temporal brain dynamics as in [25] (1 − β: 80%; α: 5%). The randomization sequence of treatment allocation was determined before the start of recruitment by random generation of blocks using the R software version 3.3.1 (R Foundation for Statistical Computing, Vienna, Austria). To ensure double-blinding of both participants and experimenters to the beverage condition, a third person (J.C.) prepared the beverages. The experimental protocol was conducted according to the Declaration of Helsinki and was approved by the Ethics Committee of the Canton de Vaud in September 2015 (protocol number 353/15). The protocol is registered in the international and national registers for clinical trials (clinicaltrials.gov: NCT02853773 and kofam.ch: SNCTP000001882). Participants were enrolled between February 2016 and April 2017. All test days were performed between March 2016 and July 2017. All experimental visits took place in the Metabolism, Nutrition and Physical Activity unit from the Center for Clinical Research of the Lausanne University Hospital. All volunteers were informed about the procedures during the screening visit and signed a written consent.

### 2.3. Meal and Test Beverage Composition

The T_0_-beverage consisted of five 70 mL-glasses (i.e., 350 mL in total) corresponding to one of the three beverage conditions (WAT, SUC, NNS). Beverage composition was based on commercialized concentrations, as determined by Ordoñez and colleagues [27]. The SUC T_0_-beverage provided 149 kcal (37.1 g of sucrose). The WAT and NNS T_0_-beverages provided 0 kcal, with 137.2 mg cyclamate, 63.35 mg acesulfame K and 40.6 mg aspartame for the NNS. The standardized meal provided at *T* = 0 min was identical for all three beverage conditions. It corresponded to 30% of the estimated individual 24-h energy requirements calculated from the Harris–Benedict equation, and was low in sugars and sweet taste (55% carbohydrates (2% sugars), 30% lipids and 15% proteins). Participants were asked to drink one 70 mL-glass every five minutes and consumed the provided meal in-between, i.e., starting and ending the meal with the consumption of a 70 mL-glass. To maximize the sweet taste receptor stimulation by sucrose or NNS, each mouthful of liquid was to be kept in the mouth for ten seconds before swallowing. The T_210_-beverage was of the same composition as the T_0_-beverages. The SUC T_210_-beverage thus provided 85 kcal (21.2 g of sucrose). The WAT and NNS T_210_-beverages provided 0 kcal, with 78.4 mg cyclamate, 36.2 mg acesulfame K and 23.2 mg aspartame for the NNS. All beverages were provided at room temperature.

### 2.4. Qualitative and Quantitative Assessments of Spontaneous Food Intake

The ad libitum buffet presented at the end of the test day comprised 12 snacks, subdivided into 4 categories (3 snacks each) based on the fat content and taste quality of the foods provided, i.e., Low Fat/Non-Sweet (LF/NSW), Low Fat/Sweet (LF/SW), High Fat/Non-Sweet (HF/NSW), and High Fat/Sweet (HF/SW). The threshold for Low-Fat/High-Fat and Non-Sweet/Sweet subdivisions was set at 10 g of lipids/sugars per 100 g of food based on the nutritional information available on the packaging. The textures were matched as much as possible between food categories. The presentation context was kept as identical as possible between all beverage conditions, and always took place in the kitchen of the Clinical Research Center. Snacks were consistently prepared by the same experimenters (C.C. and L.C.), weighed and presented following the same protocol, and served on identical white dishes. Each snack was available in larger quantity than the expected average intake. The environment was kept neutral (e.g., no visible food packaging and no particular odor), and each participant was either accompanied by the experimenter or left alone for periods of five minutes. Participants remained uninformed of their food intake being measured, and were told to eat until feeling comfortably sated. Questions regarding food type were answered, but no nutritional information was given. Water was provided ad libitum with the snacks. All snack leftovers were carefully weighed after consumption. Total energy intake and energy intake per food category was calculated based on the nutritional information available on the packaging.

### 2.5. Analytical Procedures for Plasma Samples

Plasma was separated from blood cells immediately after sampling by centrifugation during 10 min at 4 °C and 3500 rotations per minute. Aliquots of plasma were stored at −20 °C until analysis. Plasma glucose concentrations were measured by enzymatic methods (Randox Laboratories Ltd., Crumlin, UK). Plasma insulin and ghrelin concentrations were determined by radioimmunoassay (Merck Millipore Merck KGaA, Darmstadt, Germany).

### 2.6. Behavioral Ratings

VAS for hunger, thirst, and satiety consisted of 15-cm long lines with ‘0′ and ‘100′ anchored to the left and right side, respectively, presented with the written indication “*Please indicate how hungry/thirsty/satiated you are at present by drawing a point on the line below*”. Individuals’ responses were converted to % of the scale maximum. Taste cravings were assessed with a 9-point LS with ‘salty’ and ‘sweet’ anchored to the left and right side, presented with the written indication “*Please indicate how much you crave for a rather salty or sweet food item at present by ticking the correct box on the scale below*”.

### 2.7. Statistical Analyses of Food Intake, Behavioral Ratings and Plasmatic Parameters

Data distribution was controlled for normality and homoscedasticity using the Shapiro–Wilk and Bartlett tests, respectively. Data that were not normally distributed were transformed using the BoxCox algorithm. All anthropometric parameters (body weight, body mass index (BMI), and body composition), plasma concentrations and behavioral parameters (VAS and LS ratings) were tested for differences between beverage condition baselines with a one-way repeated-measure ANOVA including the independent within-subject factor of Beverage (three levels: WAT, SUC, and NNS). The impact of beverage type on food intake at the ad libitum buffet (total energy intake) was investigated first by a one-way repeated-measure ANOVA with the within-subject factor of Beverage. In a second step, the impact of the beverage type was detailed for food categories ingested by a two-way repeated-measure ANOVA with the within-subject factors of Beverage and Food category (four levels: LF/NSW, LF/SW, HF/NSW and HF/SW). Whenever a significant main effect of Beverage or an interaction Beverage × Food category was found, post-hoc paired *t*-tests (two-tailed) were conducted between Beverage conditions and/or Food categories. The effect of beverage type on the kinetics of plasma concentrations and behavioral scales was assessed using two-way repeated-measure ANOVAs with the within-subject factors of Beverage and Time. Whenever an interaction Beverage × Time was found, post-hoc paired *t*-tests (two-tailed) were conducted between Beverage conditions at each time point. Further, one-way repeated-measure ANOVAs with the within-subject factor of Beverage were conducted on plasma concentrations and behavioral scales at *T* = 210 min, irrespective of the kinetic results, to investigate the pre-buffet state. Whenever a main effect of Beverage was found, post-hoc paired *t*-tests (two-tailed) were conducted between beverage conditions. All data are expressed as mean ± SEM (standard error of mean). All analyses were performed with R version 3.3.1 (R Foundation for Statistical Computing, Vienna, Austria), and *p*-values ≤ 0.05 were considered as significant.

### 2.8. Electroencephalography (EEG) Stimuli Presentation Procedure, EEG Acquisition and Preprocessing

During both EEG recording sessions, color photographs showing foods that differed in fat content and in taste quality were presented to participants. This image database (240 items) has been used in several studies investigating food perception [25,28,29,30] and pictures were controlled for low-level visual features [31]. Stimulus presentation took place in a light-proof room, using the E-prime 2 software (Psychology Software Tools, Inc., Pittsburgh, PA, USA). Images were presented centrally for 500 ms each on a 19″ computer screen, in 3 consecutive blocks lasting 5 min. Each block contained 120 items, i.e., 80 initial encounters and 40 repeated items. Participants were asked to categorize initial from repeated image encounters via button-press. This behavioral task served to ensure participants’ attention to food images, and they were instructed to perform as quickly and accurately as possible. Following participants’ button press, the Inter-Trial-Interval (ITI) randomly varied between 250 and 750 ms to avoid anticipatory responses. During the ITI, a fixation cross was centrally displayed on screen to avoid eye movements. Number of trials between initial and repeated items were controlled across blocks and recording sessions to ensure similar difficulty of the recognition task.

Continuous EEG was recorded while participants viewed the images and performed the online recognition task. EEG was acquired at a sampling rate of 500 Hz using a 64-channel BrainProducts ActiCAP system. Details of the electrode montage can be found on the BrainProducts website (http://www.brainproducts.com/products_apps.php). All pre-processing analyses were performed using the CarTool software version 3.51 (2268) (https://sites.google.com/site/fbmlab/cartool). Only the responses to initially encountered images were used to compute visual evoked potentials (VEPs). VEPs were computed over the period from −100 ms to +500 ms peri-stimulus epoch for each image. During single subject averaging, EEG epochs were cleaned from artifacts with a semi-automatic procedure using a 80 μV rejection criterion and visual trial-by-trial inspection. Epochs containing eye blinks or other motor artifacts were manually removed. During averaging, baseline correction was applied on the peri-stimulus period (i.e., −100 ms to +500 ms), and data was band-pass filtered at 0.1–30 Hz. An additional notch filter of 50 Hz was applied. First, VEPs were averaged for each single subject, recording session (Pre- and Post-prandial) and beverage condition (WAT, SUC, and NNS). Electrodes with artefactual signals were then interpolated [32]. In a second step, group-average VEPs were calculated for each session and beverage condition, while baseline-correcting over the pre-stimulus period and recalculating the VEPs to an average reference [33].

### 2.9. EEG Analyses and Estimations of Neural Source Activity

Time windows of interest in brain responses to food viewing in the SUC, WAT, and NNS conditions were determined around GFP peaks in the group-average Global Field Power (GFP) waveform. The GFP is a reference-independent measure of the global amplitude of the electric field (VEPs), i.e., calculated as the standard deviation of the electric field amplitude across all 64 electrodes at a given time point [34]. These peak periods in the GFP represent the time windows of highest synchronized neural activity underlying distinct steps in sensory and cognitive processes, and thus served as a rationale for further investigation of beverage type-induced modulations in source activity [25,35]. The center and width of each time windows of interest were determined by the average peak timing and the standard deviation across participants’ individual GFP peaks, across sessions and beverage conditions.

Over each time window of interest, we estimated the neural source activity based on the head-surface recorded VEPs using a local autoregressive average (LAURA) distributed a linear inverse solution [36]. That is, mean amplitudes of neural activity were calculated for each of the 4350 solution points of an inverse solution matrix based on a realistic 3D head model. The output of this algorithm is one scalar value (µA/mm^3^) per solution point, per viewing condition and time window. As the goal of our study was to investigate the differential effects of three beverages on the meal-induced modulations in brain responses to food viewing, we focused our analyses on the relative change in neural activity from pre- to post-prandial recording sessions [25].

For each time window of interest, statistical analyses first comprised of whole-brain repeated-measure one-way ANOVAs with the within-subject factor of Beverage, computed on the % neural activity change from pre- to post-prandial session on each node of the 4350 solution point matrix. Only regions showing a significant main effect of Beverage in a cluster of ≥10 neighboring nodes were considered for post-hoc region-of-interest (ROI) analyses. Results of these analyses were rendered on the Montreal Institute template brain (MNI) and Talairach coordinates of the node showing the maximal statistical difference between beverage conditions are given for each statistically determined brain region. In each ROI showing a significant main effect of Beverage, neural activity of the source node revealing maximal statistical differences plus the 6 neighboring nodes was extracted and averaged in each individuals’ data, for each beverage condition. These results are visualized as bar plots, indicating pre-to-post changes in neural activity to food viewing. Statistical outliers (< or >3 standard deviations from the mean) were removed from further analyses. Post-hoc paired *t*-tests (two-tailed) were then conducted on the respective pre-to-post change in neural activity (in %) between beverage conditions. Additionally, orthogonal one-sample *t*-tests (two-tailed) assessed, within each ROI and in each beverage condition, whether the relative pre-to-post % change in signal significantly differed from baseline (i.e., pre-prandial activity; [25]). Overall, only results with *p* ≤ 0.05 were considered significant. All analyses were conducted using customized Python scripts, the software R version 3.3.1, and the STEN toolbox version 2.0 developed by Jean-François Knebel and Michael Notter (http://doi.org/10.5281/zenodo.1164038).

## 3. Results

Participants’ body weight, BMI, body composition, plasma concentrations of glucose, insulin and ghrelin and behavioral ratings for hunger, thirst, satiety, and taste cravings are shown in Table 1 for each beverage condition. None of the parameters showed differences between beverage conditions at baseline (all *p* = ns).

### 3.1. Spontaneous Food Intake at the Ad Libitum Buffet

Total energy intake and energy intake by food category at the ad libitum buffet are shown in Table 2. Regarding total energy intake, we observed a main effect of Beverage (F_2,17_ = 3.62; *p* < 0.05), i.e., participants on average ingested significantly less energy in SUC than in WAT (Δ = −151 ± 59 kcal; t_17_ = −2.58; *p* < 0.05) and NNS (Δ = −126 ± 56 kcal; t_17_ = −2.26; *p* < 0.05). However, no significant difference was observed between WAT and NNS (Δ = 25 ± 66 kcal; t_17_ = 0.38; *p* = ns). Further analyses on energy intake segregated by food categories did not show an interaction between Beverage × Food category (F_2,17_ = 0.54; *p* = ns), i.e., participants did not modify their food choice pattern as a function of the beverage type ingested.

### 3.2. Plasma Concentrations of Metabolites and Gastro-Intestinal Hormones

**Figure 2:**
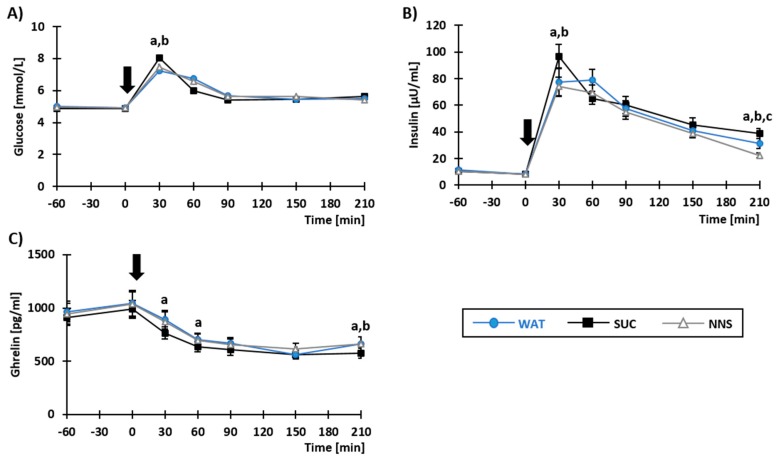
Plasma concentrations of glucose (**A**), insulin (**B**), and ghrelin (**C**) in response to beverage and concomitant meal ingestion at *T* = 0 min, indicated by a black arrow. Data are presented as mean ± SEM. ^a,b,c^: *p* < 0.05 for post-hoc paired *t*-tests (two-tailed), respectively between SUC-WAT, SUC-NNS, and WAT-NNS. WAT, SUC, NNS: Water-, Sucrose-, NNS-beverage conditions.

Plasma concentrations of glucose, insulin, and ghrelin in response to meal and test beverage ingestion are shown in Figure 2. The main effect of Beverage was significant for insulin (Figure 2B; F_2,17_ = 8.29; *p* < 0.05; i.e., plasma insulin yielded overall higher values in SUC as compared with both WAT and NNS) and ghrelin (Figure 2C; F_2,15_ = 4.56; *p* < 0.05; i.e., plasma ghrelin yielded overall lower values in SUC as compared with both WAT and NNS), but not for plasma glucose (Figure 2A; F_2,17_ = 0.15; *p* = ns). More importantly, a significant interaction between Beverage × Time was observed for all parameters (glucose: F_2,17_ = 2.83; insulin: F_2,17_ = 5.31; ghrelin: F_2,15_ = 2.41; all *p* < 0.05). Plasma glucose and insulin concentrations were significantly higher in SUC at *T* = 30 min (glucose: t_17_ = −3.77 and t_17_= −2.46; insulin: t_17_ = −3.94 and t_17_ = −5.11; all *p* < 0.05) as compared to WAT and NNS, respectively. Plasma ghrelin concentration, on the other hand, was significantly lower in SUC, as compared to WAT; this difference being significant at *T* = 30 min (t_15_ = −2.77; *p* < 0.05) and *T* = 60 min (t_15_ = −2.48; *p* < 0.05). No differences were observed between WAT and NNS for any of the parameter kinetics.

Before the buffet (*T* = 210 min), plasma glucose concentrations were similar in all beverage conditions (F_2,17_ = 0.70; *p* = ns). By contrast, a significant main effect of Beverage was observed for plasma insulin (F_2,17_ = 13.04; *p* < 0.05) and ghrelin concentrations (F_2,16_ = 9.44; *p* < 0.05). Plasma insulin concentration was most elevated in SUC (t_17_ = 2.55 and t_17_ = 4.77; both *p* < 0.05 against WAT and NNS, respectively), and the lowest in NNS (t_17_ = 2.74; *p* < 0.05 against WAT). Plasma ghrelin concentration was lower in SUC as compared with WAT (t_17_ = 2.88; *p* < 0.05) and NNS (t_17_ = 3.89; *p* < 0.05), but there was no difference between WAT and NNS (t_17_ = 0.81; *p* = ns).

### 3.3. Results of Behavioral Ratings

No main effect of Beverage nor interaction between Beverage × Time were observed for any of the parameter kinetics (Supplementary Figure S1A–D for hunger, satiety, thirst ratings and taste cravings).

Before the buffet (*T* = 210 min), a significant main effect of Beverage was found on hunger ratings (F_2,17_ = 5.68; *p* < 0.05). Hunger ratings were lower in SUC as compared with WAT (t_17_ = −2.71; *p* < 0.05) and NNS (t_17_ = −2.66; *p* < 0.05). No difference in hunger ratings was found between WAT and NNS (t_17_ = 0.28; *p* = ns). No significant main effect of Beverage was found for thirst ratings (F_2,17_ = 0.07), satiety ratings (F_2,17_ = 1.10) and taste cravings (F_2,17_ = 0.37; all *p* = ns). 

### 3.4. Pre- to Post-Prandial Changes in Neural Source Activity To Food Viewing

**Figure 3:**
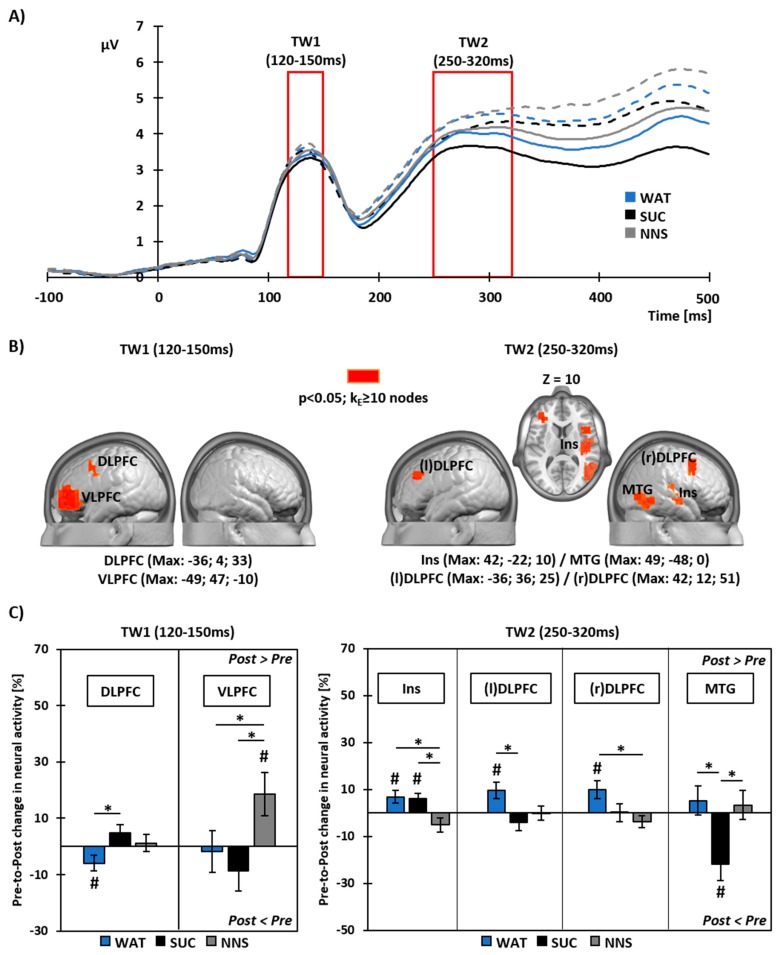
(**A**) Group-average Global Field Power (GFP) waveform over the peri-stimulus period (−100 to +500 ms from image onset). Red borders indicate the time windows of interest (TW) for subsequent neural source analyses. Solid lines illustrate the GFP during the pre-prandial recording session and dotted lines show GFP during the post-prandial recording session. (**B**) Visualization of brain regions showing a main effect of Beverage in the whole brain analyses on pre- to post-prandial changes in neural activity. Talairach coordinates (*x*, *y*, *z*) indicate the position of the source node showing maximal statistical differences. (**C**) Results of post-hoc analyses on changes in neural activity within each region of interest. Bar plots detail the direction of changes in each region of (**B**) and for each Beverage condition. Data are shown as mean (±SEM). *: *p* < 0.05 for paired *t*-tests (two-tailed) between Beverage conditions. ^#^: *p* < 0.05 for one-sample *t*-tests vs baseline (pre-prandial). VLPFC: ventrolateral prefrontal cortex. Ins: insula. (l)- & (r)DLPFC: left & right dorsolateral prefrontal cortex. MTG: Middle Temporal Gyrus. WAT, SUC, NNS: Water-, Sucrose-, NNS-beverage conditions.

Two time periods of interest were defined around the peaks of the group-average GFP waveform. A first period of interest ranged from 120 to 150 ms, and a second period of interest ranged from 250 to 320 ms after image onset (Figure 3A).

Over the first time window of interest (TW1: 120–150 ms post-image onset), whole brain analyses revealed a main effect of Beverage on the pre- to post-prandial % change in neural activity in the left dorsolateral prefrontal cortex (DLPFC; Max: *x* = −36, *y* = 4, *z* = 33) and in the left ventrolateral prefrontal cortex (VLPFC; Max: *x* = −49, *y* = 47, *z* = −10). That is, the beverage type differentially modulated the neural activity to food viewing within these brain areas (Figure 3B, left panel).

With WAT, meal intake led to decreased neural activity within the DLPFC (t_16_ = −2.16; *p* < 0.05 for *t*-test against baseline), but did not impact neural activity within the VLPFC (t_17_ = −0.25; *p* = ns for *t*-test against baseline) (Figure 3C, left panel). Unlike WAT, there was no modulation in the neural response within the DLPFC with SUC (t_16_ = 1.66; *p* = ns for *t*-test against baseline; t_16_ = 3.31; *p* < 0.05 for paired *t*-test between WAT and SUC responses). Like WAT however, SUC did not lead to modulated neural activity within the VLPFC (t_17_ = −1.22; *p* = ns for *t*-test against baseline). NNS also did not impact neural activity within the DLPFC (t_16_ = 0.39; *p* = ns). Yet, in contrast to WAT and SUC, NNS led to increased neural activity within the VLPFC (t_17_ = 2.42; *p* < 0.05 for *t*-test against baseline; t_17_ = −3.20 and t_17_ = −4.19; both *p* < 0.05 for paired *t*-tests between NNS-WAT and NNS-SUC, respectively).

Over the second time window of interest (250–320 ms post-image onset), a main effect of Beverage on the pre- to post-prandial % change in neural activity was observed in the right insula (Ins; Max: *x* = 42, *y* = −22, *z* = 10), in the left (l) and right (r) DLPFC ((l)DLPFC Max: *x* = −36, *y* = 36, *z* = 25 and (r)DLPFC Max: *x* = 42, *y* = 12, *z* = 51), and in the right middle temporal gyrus (MTG; Max; *x* = 49, *y* = −48, *z* = 0) (Figure 3B, right panel).

With WAT, meal intake led to increased neural activity within the insula (t_17_ = 2.55; *p* < 0.05), (l)DLPFC (t_17_ = 2.82; *p* < 0.05) and (r)DLPFC (t_16_ = 2.60; *p* < 0.05), but did not impact neural activity within the MTG (t_16_ = 0.85; *p* = ns; all *t*-tests against baseline) (Figure 3C, right panel). Like WAT, SUC also led to increased neural activity within the insula (t_17_ = 2.48; *p* < 0.05 for *t*-test against baseline). Unlike WAT however, there were no pre-to-post changes in neural activity in SUC in the (l)DLPFC (t_17_ = −1.09; *p* = ns for *t*-test against baseline; t_17_ = −4.94; *p* < 0.05 for paired *t*-test between SUC and WAT) and the (r)DLPFC (t_16_ = 0.04; *p* = ns for *t*-test against baseline). In addition, SUC led to decreased neural activity within the MTG (t_16_ = −3.21; *p* < 0.05 for *t*-test against baseline; t_16_ = −3.74; *p* < 0.05 for paired *t*-test between SUC and WAT). In contrast to WAT and SUC, NNS did not impact neural activity within the insula (t_17_ = −1.72; *p* = ns for *t*-test against baseline; t_17_ = 3.11 and t_17_ = 2.86; both *p* < 0.05 for paired *t*-tests between NNS-WAT and NNS-SUC, respectively). Like in SUC, there were no pre-to-post changes in neural activity in NNS within the (l)DLPFC (t_17_ = −0.01; *p* = ns for *t*-test against baseline) and the (r)DLPFC (t_16_ = −1.49; *p* = ns for *t*-test against baseline; t_16_ = 2.56; *p* < 0.05 for paired *t*-test between NNS and WAT). Like WAT, but unlike SUC, NNS consumption did not impact neural activity within the MTG (t_16_ = 0.55; *p* = ns for *t*-test against baseline; t_16_ = −2.46; *p* < 0.05 for paired *t*-test between NNS and SUC).

## 4. Discussion

Our study aimed at investigating the acute impact of consuming caloric (sucrose) and non-caloric sweeteners (NNS), as compared to water, on the subsequent brain responses to visual food cues and spontaneous food intake behavior. As expected, we found neurophysiological and physiological markers of satiety following the ingestion of the standardized meal with water. We further observed that sucrose consumption impacted the responses in brain areas associated with cognitive control (prefrontal cortices) and food categorization (temporal cortices), and led to decreased subsequent food intake, indicating an adequate compensatory behavior. In contrast, NNS consumption did not alter spontaneous food intake when compared to water, but altered postprandial brain responses to visual food cues, most pronounced in prefrontal areas and in the insula.

### 4.1. Brain Responses to Food Viewing Following Water or Sucrose Consumption

Meal ingestion combined with water (i.e., control beverage condition lacking sweet taste and caloric load) impacted brain responses to visual food cues in bilateral dorsal prefrontal areas and in the right insula. The neural activity in dorsal prefrontal areas has long been linked with the capacity to exert cognitive control over food intake when exposed to palatable food cues, as part of the executive function network. Tataranni and colleagues [37] were the first to highlight differences in brain responses between hunger and satiety beyond hypothalamic areas using functional neuroimaging, and found increased neural activity in the dorsolateral prefrontal cortex. Since then, many other studies have found dorsolateral prefrontal regions to be involved in top-down cognitive control over food intake [38,39,40]. A study of Camus and colleagues [41] could even attest causality in the role of dorsolateral prefrontal regions in control and decision-making using transcranial magnetic stimulation. Using the high temporal resolution of EEG, Harris and colleagues [42] were able to provide further insights on the dual role of DLPFC in cognitive control, showing that early response modulations (around 150 ms post-stimulus onset) were associated with top-down filtering of sensory input, whereas later ones (from 450 ms post-stimulus onset) were associated with reward value modulation. In our study, we observed decreased activity in the left dorsal prefrontal region over an early time window following food viewing (120–150 ms) and increased activity in the bilateral dorsal prefrontal region over later timing (250–320 ms). Our findings thus likely reflect elevated cognitive control following meal ingestion, which is rather due to value integration than to response modulation by the sensory input per se.

We also observed increases in neural activity in the insula following meal ingestion accompanied by water. Insular responses to visual food cues have consistently been associated with interoception, i.e., awareness of bodily energy states. Also, the insula does contain molecular receptors for several gastro-intestinal hormones relaying this peripheral information to central nervous responses [11,43,44]. Furthermore, other studies have found increased insular activity subsequent to PYY infusion mimicking satiety [45], to mouth rinsing with a glucose drink mimicking food intake anticipation [46], but also in response to calorie ingestion as such [20,47]. The insula, being a hub between salience, homeostatic and control networks, is generally involved in signal integration, and thought to perform flavor-nutrient conditioning, too [48]. In accordance with these findings, we also found a higher postprandial insular activity following sucrose ingestion. Altogether, increases in insular activity both in the sucrose and water conditions thus likely reflect the adequate adaptation of participants’ responses as a function of the beverage consumed when taste properties and caloric load were congruent.

Sucrose drinking (i.e., the beverage condition combining sweet taste and caloric load) elicited partially different modulations in brain responses to visual food cues as compared to meal ingestion with water. In particular, the postprandial response to visual food cues in cognitive control related areas was found blunted in the sucrose condition. Sucrose consumption also led to markedly decreased neural activity in the middle temporal lobe. This brain area is involved in the categorization and optimization of visual stimulus processing by attention [49], and is generally more active when participants are exposed to palatable food over neutral stimuli [50], as well as when responses to food are compared between hunger and satiety [24,51]. These differential patterns of brain responses to food cues following sucrose *vs.* water ingestion likely show that when sweet taste is coupled to a caloric load, brain responses shift from a rather reflective (usually involving prefrontal brain areas) to a more reflexive processing of food cues [52].

### 4.2. Brain Responses to Food Viewing Following NNS Consumption

NNS consumption (i.e., the beverage condition with discrepant sweet taste and caloric load) also yielded differential neural activity in response to subsequent exposure to visual food cues. In contrast to the ingestion of the meal with water or a sucrose drink, we observed early enhanced ventrolateral prefrontal cortex activity (120–150 ms after food image onset), but no changes in insular activity to food viewing over the later time window of interest (250–320 ms).

Increases in neural activity following NNS tasting in ventrolateral prefrontal cortices has been highlighted in gustatory processing when neural responses were assessed at the time of or immediately after tasting. For instance, Smeets and colleagues [53] have shown greater activation of the ventrolateral prefrontal cortex directly after the ingestion of an artificially sweetened beverage as compared to sucrose. Ventral prefrontal regions have been widely associated with hedonic integration and reward valuation of (visually) perceived stimuli, including food cues [54,55,56]. However, these functions were mostly attributed to medial parts of the ventral prefrontal cortex, whereas we show enhanced activity within the ventrolateral prefrontal cortex in response to visual cues following NNS consumption. Ventrolateral regions of the prefrontal cortex are part of the executive function circuitry, supporting decision-making adjustments, in particular related to motor response inhibition when exposed to cues associated with high reward, as well as targeting attention to behaviorally significant stimuli (reviewed in [57]). Thus, this area is proposed to be responsible for altering behavior as a function of estimated changes in the reward value of (viewed) stimuli. Our results show increased neural activity to visual food cues within the ventrolateral prefrontal area, likely related to greater (need for) impulse retaining and control over anticipated food intake. Although no study so far investigated the impact of NNS consumption on brain responses to food cues longitudinally, research in the gustatory modality showed differences in neural activation to NNS tasting between non-diet soda drinkers and frequent consumers of diet soda [58]. The study of Green & Murphy found increased responses to saccharin vs. sucrose in the ventrolateral prefrontal cortex in non-diet soda drinkers, whereas this difference was absent in frequent diet soda drinkers. These findings were interpreted as reflecting ‘fading’ neural activity in this region with repeated consumption of NNS, impacting impulse control over time. In line, we previously found decreased activity in the more posterior part of the ventrolateral prefrontal cortex following a 3-month replacement of SSBs by non-calorically sweetened equivalents [25]. Our current results thus provide additional evidence as to a target region for future longitudinal studies on the longer-term impact of sweet taste stimulation by NNS, and on responses to tempting visual food cues following NNS ingestion.

With NNS consumption, on the other hand, we did not observe pre-to postprandial changes in insular activity. This suggests that congruent caloric and taste signaling is required to elicit adequate response adaptation to food cues, and that incongruences between taste information and caloric load may impair nutrient-flavor conditioning [48]. Rudenga and Small [59] have further shown that the neural response to sucrose tasting in the insula (and also in the amygdala) decreased as a function of NNS consumption habits of participants, implying that this region might be more vulnerable to chronic dissociations between sweet taste signaling and metabolic consequences.

### 4.3. Integration of Postprandial Brain Responses to Food Viewing with Gastro-Intestinal Hormone Secretion and Food Intake Behavior

Sucrose drinking during meal ingestion, as compared to water, led to subsequent decreased food intake at the ad libitum buffet indicative of a compensatory food intake behavior. In parallel, we observed elevated plasma concentrations of insulin (an anorexigenic hormone) and decreased plasma concentrations of ghrelin (an orexigenic hormone), likely promoting some of the brain response alterations. Thus, the effects of sucrose intake may be related to hormonal signaling and/or to sweet taste receptor activation coupled with other peripheral satiety signals (e.g., vagal afferents). Yet, whether the observed differences are sucrose-specific effects or more general ones driven by an extra caloric load cannot be concluded from our current study, as there was no condition with a caloric load from another nutrient source (e.g., maltodextrin or fat).

NNS consumption did not lead to pronounced modulations of glucose, insulin, and ghrelin concentrations, nor to higher caloric consumption or variation of the food choice pattern at the ad libitum buffet. Thus, the observed changes in brain activity to food viewing post-meal and food intake pattern cannot be attributed solely to differential signaling of gastro-intestinal mediators. Although we did not measure other anorexigenic hormones such as leptin or PYY, the observed differences in brain responses between the water and NNS condition are congruent with the idea that discrepant information between sweet taste receptor activation and gastrointestinal hormone signaling leads to changes in brain response patterns [12].

### 4.4. Limitations

Several limitations of our work need to be considered. First, the study design likely pronounces the impact of the meal ingestion stronger than the impact of the test beverage. However, we aimed at designing this study with the highest ecological validity, i.e., having volunteers consuming standard amounts of beverages concomitant with a meal (quantity close to a commercially available can size). For this reason, we cannot exclude that the design was not sensitive enough to detect all secondary outcome differences, especially between the water and NNS conditions, and in terms of qualitative analyses on food choice patterns. Second, while we used a double-blinded design, participants could still detect the absence of sweet taste in the water condition, as opposed to both sweet taste conditions. Thus, some differences in brain response patterns might have arisen from these perceptual properties [60]. Finally, using electroencephalographic recording and electrical neuroimaging analyses, we are not able to detect deeper activity changes, e.g., in the basal ganglia (dopaminergic origin of the reward system), that might occur together with response modulations in cortices associated with higher-level functions.

## 5. Conclusions

To our knowledge, this is the first study to assess the impact of NNS consumption on neural activity to food viewing, and the relationship with food intake behavior. We did not observe an acute effect of NNS consumption on immediate food intake in humans who are not frequently drinking NNS beverages. Yet, we observed imminent changes in brain response patterns in brain areas that are key players in food intake regulation. The responsiveness of these brain areas to sweet taste has been shown to ‘fade’ as a function of longer-term NNS consumption [58,59]. Thus, it remains to be investigated whether such longer-term brain response alterations can also be observed to visual food cues, often mediating pre-ingestive food choices. Given such longer-term alterations, the brain response modulations observed under the NNS condition in our study might reflect an initial stage of adaptation to taste-calorie uncoupling, possibly indicating that longer-term alterations of food intake regulation (via responses to tempting visual cues) take place when NNS are repeatedly consumed over time. Our study thus provides first insights linking neuroimaging research in the gustatory modality and behavioral research on the impact of non-caloric sweetener consumption on food intake, by investigating the neural correlates of drives towards visually conveyed food cues.

## Figures and Tables

**Table 1:** Study participants’ anthropometric, metabolic, and behavioral characteristics at baseline.

	Beverage Condition			One-Way ANOVA	
	WAT	SUC	NNS	F-Value	*p*-Value
Body weight [kg]	66.6 ± 1.2	66.5 ± 1.2	66.5 ± 1.1	0.39	0.68
BMI [kg/m^2^]	21.4 ± 0.4	21.4 ± 0.4	21.3 ± 0.4	0.12	0.89
Body fat mass [kg]	12.3 ± 1.2	12.2 ± 0.9	12.2 ± 1.1	0.04	0.97
Plasma glucose [mmol/L]	4.9 ± 0.1	4.8 ± 0.1	4.9 ± 0.1	1.23	0.31
Plasma insulin [µU/mL]	10.3 ± 0.7	9.8 ± 0.5	9.5 ± 0.7	2.33	0.11
Plasma ghrelin [pg/mL]	984.8 ± 94.1	925.7 ± 70.4	963.3 ± 94.1	0.90	0.41
Hunger level [%]	71.4 ± 5.1	73.3 ± 4.4	72.6 ± 4.7	0.12	0.89
Thirst level [%]	55.7 ± 5.5	67.1 ± 3.8	61.5 ± 5.1	2.38	0.11
Satiety level [%]	21.7 ± 4.2	23.6 ± 4.8	20.4 ± 3.4	0.25	0.78
Taste cravings (1–9)	5.4 ± 0.4	5.6 ± 0.5	5.8 ± 0.5	0.30	0.75

Data are expressed as mean ± SEM (standard error of mean). WAT, SUC, NNS: Water-, Sucrose-, NNS-beverage conditions. BMI: body mass index.

**Table 2:** Spontaneous food intake at the ad libitum buffet.

	Beverage Condition		
	WAT	SUC	NNS
Total energy intake [kcal]	942 ± 71	791 ± 62 ^a,b^	917 ± 70
Energy intake from LF/NSW foods [kcal]	142 ± 28	141 ± 29	167 ± 37
Energy intake from LF/SW foods [kcal]	77 ± 22	62 ± 15	78 ± 20
Energy intake from HF/NSW foods [kcal]	515 ± 74	428 ± 50	449 ± 56
Energy intake from HF/SW foods [kcal]	209 ± 36	161 ± 24	224 ± 35

Data are expressed as mean ± SEM. WAT, SUC, NNS: Water-, Sucrose-, NNS-beverage conditions. LF, HF: low-, high-fat. NSW, SW: Non-sweet, sweet. ^a^ *p* < 0.05 for post-hoc paired *t*-test (two-tailed) between SUC and WAT. ^b^ *p* < 0.05 for post-hoc paired *t*-test (two-tailed) between SUC and NNS.

## Supplementary Materials

The following materials are available online at http://www.mdpi.com/2072-6643/10/5/615/s1. Figure S1: Behavioral ratings for hunger, satiety, thirst, and taste cravings in response to drink and concomitant meal ingestion.
