# Supplementary material for: The Impact of Caloric and Non-Caloric Sweeteners on Food Intake and Brain Responses to Food: A Randomized Crossover Controlled Trial in Healthy Humans
Source: Nutrients. 2018 May 15;10(5):615. doi: 10.3390/nu10050615 (PMC5986495; doi:10.3390/nu10050615)
Supplement: Supplementary file 1 [file nutrients-10-00615-s001.pdf]

Figure S1

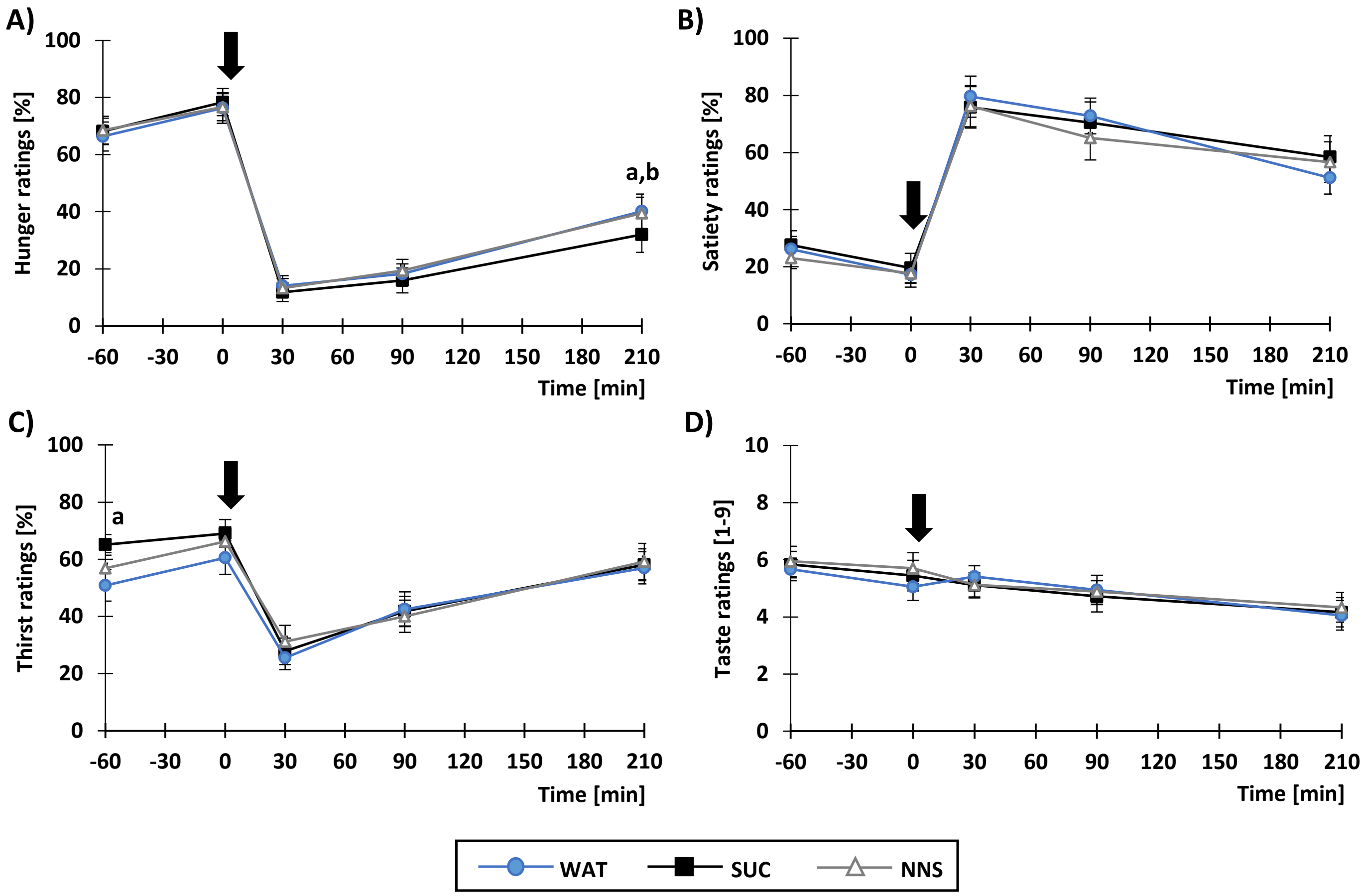

Figure S1. Behavioral ratings for hunger **(A)**, satiety **(B)**, thirst **(C)** and taste cravings **(D)** in response to drink and concomitant standardized meal ingestion at *T* = 0 minute, indicated by a black arrow. A taste rating of 1 indicates the maximum preference for a salty item whereas 9 indicates the maximum preference for a sweet item. Data are presented as mean ± SEM. [a, b]: $p<0.05$ for post-hoc paired t-tests (two-tailed), respectively between SUC-WAT and SUC-NNS. WAT, SUC, NNS: Water-, Sucrose-, NNS-beverage conditions.
